# Detection of Quiescent Radioresistant Epithelial Progenitors in the Adult Thymus

**DOI:** 10.3389/fimmu.2017.01717

**Published:** 2017-12-05

**Authors:** Maude Dumont-Lagacé, Hervé Gerbe, Tariq Daouda, Jean-Philippe Laverdure, Sylvie Brochu, Sébastien Lemieux, Étienne Gagnon, Claude Perreault

**Affiliations:** ^1^Institute for Research in Immunology and Cancer, Montreal, QC, Canada; ^2^Department of Medicine, Université de Montréal, Montréal, QC, Canada; ^3^Department of Biochemistry, Université de Montréal, Montréal, QC, Canada; ^4^Department of Informatics and Operational Research, Université de Montréal, Montréal, QC, Canada; ^5^Department of Microbiology, Infectiology and Immunology, Université de Montréal, Montréal, QC, Canada

**Keywords:** thymic epithelial cells, stem cells, thymic regeneration, label-retention assay, transcriptomics

## Abstract

Thymic aging precedes that of other organs and is initiated by the gradual loss of thymic epithelial cells (TECs). Based on *in vitro* culture and transplantation assays, recent studies have reported on the presence of thymic epithelial progenitor cells (TEPCs) in young adult mice. However, the physiological role and properties of TEPC populations reported to date remain unclear. Using an *in vivo* label-retention assay, we previously identified a population of quiescent but non-senescent TECs. The goals of this study were therefore (i) to evaluate the contribution of these quiescent TECs to thymic regeneration following irradiation-induced acute thymic injury and (ii) to characterize their phenotypic and molecular profiles using flow cytometry, immunohistology, and transcriptome sequencing. We report that while UEA1^+^ cells cycle the most in steady state, they are greatly affected by irradiation, leading to cell loss and proliferative arrest following acute thymic involution. On the opposite, the UEA1^–^ subset of quiescent TECs is radioresistant and proliferate *in situ* following acute thymic involution, thereby contributing to thymic regeneration in 28- to 30-week-old mice. UEA1^–^ quiescent TECs display an undifferentiated phenotype (co-expression of K8 and K5 cytokeratins) and express high levels of genes that regulate stem cell activity in different tissues (e.g., *Podxl* and *Ptprz1*). In addition, two features suggest that UEA1^–^ quiescent TECs occupy discrete stromal niches: (i) their preferential location in clusters adjacent to the cortico-medullary junction and (ii) their high expression of genes involved in cross talk with mesenchymal cells. The ability of UEA1^–^ quiescent TECs to participate to TEC regeneration qualifies them as *in vivo* progenitor cells particularly relevant in the context of regeneration following acute thymic injury.

## Introduction

In all vertebrates, the thymus is the sole organ that can generate functional classic (TCRαβ^+^) T lymphocytes (1). Thymic epithelial cells (TECs) are responsible for the unique properties of the thymus: they orchestrate each steps of T-cell development and regulate thymic output (2, 3). In adults, the production of naive T cells gradually decreases with age, a decline caused by the loss of TECs, which entails a reduction in the TCR repertoire diversity (4). It was therefore somewhat surprising to realize that TECs are not post-mitotic cells and that they retain extensive regenerative capacities even in adults (5, 6). Indeed, medullary TECs (mTECs), and to a lesser extent cortical TECs (cTECs), turnover rapidly in healthy animals (6–8). In addition, the thymus has the ability to regenerate following various acute injuries induced by stress, infection, sublethal irradiation, or pregnancy (9–13). However, more severe injuries inflicted by chemotherapy or radiation therapy can overwhelm the regenerative potential of the thymic epithelium and lead to prolonged immune deficiency (14, 15). Since tissue repair is usually driven by stem-progenitor cells, identification of thymic epithelial progenitor cells (TEPCs) responding to thymic injury should provide key insights into the mechanisms of thymic regeneration.

Various stem cells including hematopoietic stem cells (16), muscle satellite cells (17), and hair follicle stem cells (18) are quiescent in steady-state conditions. We therefore hypothesized that isolation of non-dividing TECs might enable us to enrich for a population of quiescent TEPCs. To this end, we used an *in vivo* label-retaining cell (LRC) assay in which cells expressed a histone 2B-GFP fusion protein (H2B-GFP) inducible under the control of the reverse tetracycline-controlled transactivator (rtTA). Using this approach, we have previously shown that in adult mice, the UEA1^–^ TECs (mostly cTECs) contain more LRCs than UEA1^+^ TECs (mTECs), and that non-dividing UEA1^–^ LRCs were quiescent rather than senescent (7). Indeed, UEA1^–^ LRCs expressed low levels of senescence-associated transcripts (*p16INK4, p19ARF*, and *Serpine1*) and high levels of transcripts instrumental to TEC regeneration (*Bmi1, Trp63*, and *Wnt4*).

Previous studies have shown that transplantation can activate stem cell behavior in cells that do not act as stem cells in normal situation (19). The goal of this study was therefore to evaluate the contribution of endogenous (untransplanted) LRCs to thymus regeneration and to gain further insights into their *in situ* spatial distribution and molecular attributes. In addition, we wished to evaluate the proliferative activity of TEC subsets in two settings: steady-state conditions vs thymic regeneration following acute injury. We report that while the non-quiescent UEA1^+^ TECs cycle more actively than other TEC subsets under steady-state conditions, they are greatly affected by irradiation, leading to cell loss and a significant decrease in their proliferative activity. On the contrary, while other TEC subsets (i.e., UEA1^–^ TECs and quiescent UEA1^+^ TECs) proliferate modestly in physiological settings, they did not suffer cell loss from radiations. Interestingly, one particular TEC subset, the UEA1^–^ LRCs, increased its proliferation during the regenerative phase following thymic injury induced by irradiation, showing that it contains quiescent radioresistant TEPCs activated during tissue repair.

Using immunofluorescence analysis, we observed that most LRCs co-express both K8 and K5 cytokeratins, an undifferentiated phenotype observed in embryonic TEPCs, and are located near the cortico-medullary junction (CMJ) where they form cell clusters. Furthermore, the transcriptomic profile of UEA1^–^ LRCs showed low expression of genes implicated in interactions with thymocytes and high expression of genes involved in interactions with stromal cells and extracellular matrix (ECM). These results suggest that UEA1^–^ LRCs are localized in specialized niches which are instrumental in the regulation of TEPCs activity. Finally, we identified six potential regulators of quiescent radioresistant TEPCs that are known to either regulate stem cell activity through niche interactions in other tissues (*Podxl, Ptprz1*, and *Angpt1*) or whose stromal cell ligands regulate thymic output (*Tgfrb3, Fzd4*, and *Ar*).

## Materials and Methods

### Mice

B6.Cg-Gt(ROSA)26Sor^tm1(rtTA*M2)Jae^/J and STOCK Tg(tetO-HIST1H2BJ/GFP)47Efu/J mice purchased from The Jackson Laboratory (Bar Harbor) were bred and housed under specific-pathogen-free conditions in sterile ventilated racks at the Institute for Research in Immunology and Cancer. For H2B-GFP pulse-chase experiments, doxycycline was incorporated in food (2 g/kg) (Harlan Laboratories), or in drinking water (2 mg/ml of doxycycline supplemented with 5% sucrose) for a 6-week pulse period. Only female mice were analyzed in this study. All procedures were in accordance with the Canadian Council on Animal Care guidelines and approved by the Comité de Déontologie et Expérimentation Animale de l’Université de Montréal.

### Thymic Stroma Digestion

Enrichment of thymic stromal cells was performed as previously described (20). Briefly, thymic tissue was cut into small fragments and thymocytes released were removed from the supernatant. Stromal fragments were then digested at 37°C using a solution of 0.01% Liberase TM (Roche Applied Science) and 0.1% DNase-1 (Sigma-Aldrich) in RPMI-1640 with HEPES (Gibco) for three periods of 15 min. After the second incubation, cells released in the supernatant were removed and placed on ice and new fresh enzyme solution was added to the remaining fragments. The stromal cells in suspension were filtered before staining and analysis.

### Flow Cytometry and Immunofluorescence Microscopy

The list of antibodies is provided in Supplementary Experimental Procedures (Table S1 in Supplementary Material). Viability of cells was assessed using 7-AAD or Propidium Iodine (BD Biosciences). Cell sorting was performed using three laser FACSAria (BD Biosciences) or analyzed on a three laser LSR II (BD Biosciences) using FACSDiva software (BD Biosciences).

Immunofluorescence microscopy was performed on cryosections of female thymi extracted after a 16-week chase period. Appropriate isotype and negative controls were included in all experiments. For detection of immunofluorescence, slices were examined using the Nanozoomer 2.0-HT from Hamamatsu, and NDPscan software (Hamamatsu) was used for image analysis. Quantification of K5^+^, K8^+^, or K5^+^K8^+^ surface area was performed using ImageJ, and LRCs were identified as having fluorescence intensity four times that of the cells in the negative controls with the maximal fluorescence intensity (see Figure S1 in Supplementary Material).

### Thymic Injury and Bromodeoxyuridine (BrdU) Incorporation

Female mice at 16 weeks of chase were irradiated at a sublethal dose of 550 cGy to induce thymic involution (day 0). Four days after the irradiation, intraperitoneal injections of BrdU (1.5 mg per injection) were given daily for 3 days (days 4–6). At day 7, the mice were sacrificed, and the thymus was extracted for analysis. For control mice, the same procedure was performed without irradiation, at the end of the chase period.

### Statistical Analyses

Unless stated otherwise, results are expressed as means ± SD, and statistical significance was assessed using unpaired two-tailed Student’s *t*-test. We verified the goodness of fit to the Poisson distribution using maximum likelihood to assess the statistical significance of dispersion index, calculated from the location of LRCs on thymic slices.

### RNA Sequencing

We analyzed the transcriptome of two populations of TECs: UEA1^–^ LRCs (GFP^hi^) and UEA1^–^ NonLRCs (GFP^–^) from females after a 6-week pulse period and a 16-week chase period. We obtained one biological replicates of each TEC population from a pool of 11 mice to get a minimum of 10^4^ cells per sample. Total RNA was isolated using Trizol™ as recommended by the manufacturer (Invitrogen), and then further purified using RNeasy Micro columns (Qiagen). Sample quality was assessed using Bioanalyzer RNA Pico chips (Agilent). Transcriptome libraries were made using the TruSeq RNA Sample Prep Kit (v2) (Illumina) following the manufacturer’s protocols. Library generation was then assessed using a Bioanalyzer platform (Agilent) and Illumina MiSeq-QC run. Then, sequencing was done using Illumina HiSeq2000 and TruSeq SBS v3 chemistry at the Institute for Research in Immunology and Cancer’s Genomics Platform. Cluster density was targeted at around 800 k clusters/mm^2^. Data were mapped to the *Mus musculus* (mm10) reference genome using the ELANDv2 alignment tool from the CASAVA 1.8.2 software (Illumina). RNA-Seq data have been deposited in GEO archives under accession number GSE94642 and are displayed in Table S3 in Supplementary Material.^1^ Analyses of RNA-sequencing data were performed using the publicly available statistical software package “R.”^2^ To remove genes that were lowly expressed in our analysis, we considered only genes that had a relative expression higher than 1 RPKM in at least one sample. Enrichment of biological functions were performed using the Gene Functional Annotation tool from DAVID bioinformatics resources^3^ [version 6.8 (21, 22)], and reduction of redundancy through semantic similarity was performed using REViGO web-based tool for gene ontology (GO) analysis (23).

RNA-Seq data for Sca1^+^ mesenchymal cells and for UEA1^+^ TECs from female mice were extracted from Patenaude and Perreault (24) and Dumont-Lagacé et al. (25), respectively. Data can be found under GEO accession numbers GSE60101 and GSE66873.

## Results

### Experimental Model

The ROSA26-rtTA;TetO-H2B-GFP transgenic mouse model allows the identification of slow-cycling cells through label retention in a pulse-chase assay (18, 26, 27). The reverse tetracycline-controlled transactivator (rtTA) allows doxycycline-inducible expression of H2B-GFP in all cells. The proliferative history of cells can therefore be evaluated by measuring the fluorescence intensity of the remaining H2B-GFP over time. After pulse, the H2B-GFP fluorescence of non-dividing cells remains above negative control (H2B-GFP^+^ WT, see Figure S2 in Supplementary Material) for at least 6 months after doxycycline withdrawal, and at least five cell divisions are required for H2B-GFP to become undistinguishable from negative control by flow cytometry (28, 29).

In adults, female TECs proliferate more actively than male TECs, mostly because of the inhibitory effect of androgens (7, 25). Therefore, it is preferable to analyze TECs from both sexes separately. In this study, we analyzed only female mice from which TECs (EpCAM^+^CD45^–^) were divided into two populations based on UEA1 expression (Figure S2A in Supplementary Material). As UEA1^+^ TECs derive from an undifferentiated UEA1^–^ progenitor (30, 31), we analyzed UEA1^+^ and UEA1^–^ TECs, reasoning that progenitor cells should be enriched in the UEA1^–^ compartment. The 6-weeks doxycycline treatment was initiated at 4–6 weeks of age (pulse) and was followed by a chase period of 16 weeks (26–28 weeks of age at time of analysis). We defined two fluorescence thresholds for experimental purposes: (i) GFP^hi^ cells, with the fluorescence intensity of LRCs, that is, cells that did not divide during the 16 weeks of chase, taking into account the 24-day half-life of the H2B-GFP protein (Figure S2B in Supplementary Material) (32) and (ii) GFP^int^ cells, with a fluorescence intensity above the negative control but below the GFP^hi^ threshold. The fluorescence intensity of LRCs (GFP^hi^) after the chase period was at least four times higher than the threshold that distinguishes GFP^–^ from GFP^int^ cells (Figure S2B in Supplementary Material). This means that at the term of the chase period, an LRC would become GFP^–^ after a minimum of three cell divisions (eightfold GFP dilution). Henceforth, in flow cytometry analyses, the term LRC will only be used to refer to GFP^hi^ cells.

Whereas the initial 6-week pulse enabled the homogenous and high GFP labeling of >75% of TECs, only a small proportion of TECs remains GFP^hi^ (i.e., LRCs) after 16 weeks of chase: 5.5% of UEA1^–^ TECs and 1.8% of UEA1^+^ (Figures S2B,C in Supplementary Material). Approximately 60% of LRCs were UEA1^–^, even if only one-third of TECs are UEA1^–^ (Figure S2C in Supplementary Material) This is consistent with previous studies showing that mTECs (UEA1^+^) turnover more rapidly than cTECs (UEA1^–^) in adult mice (7, 25).

### Most LRCs Cluster in Proximity to the CMJ

**Figure 1:**
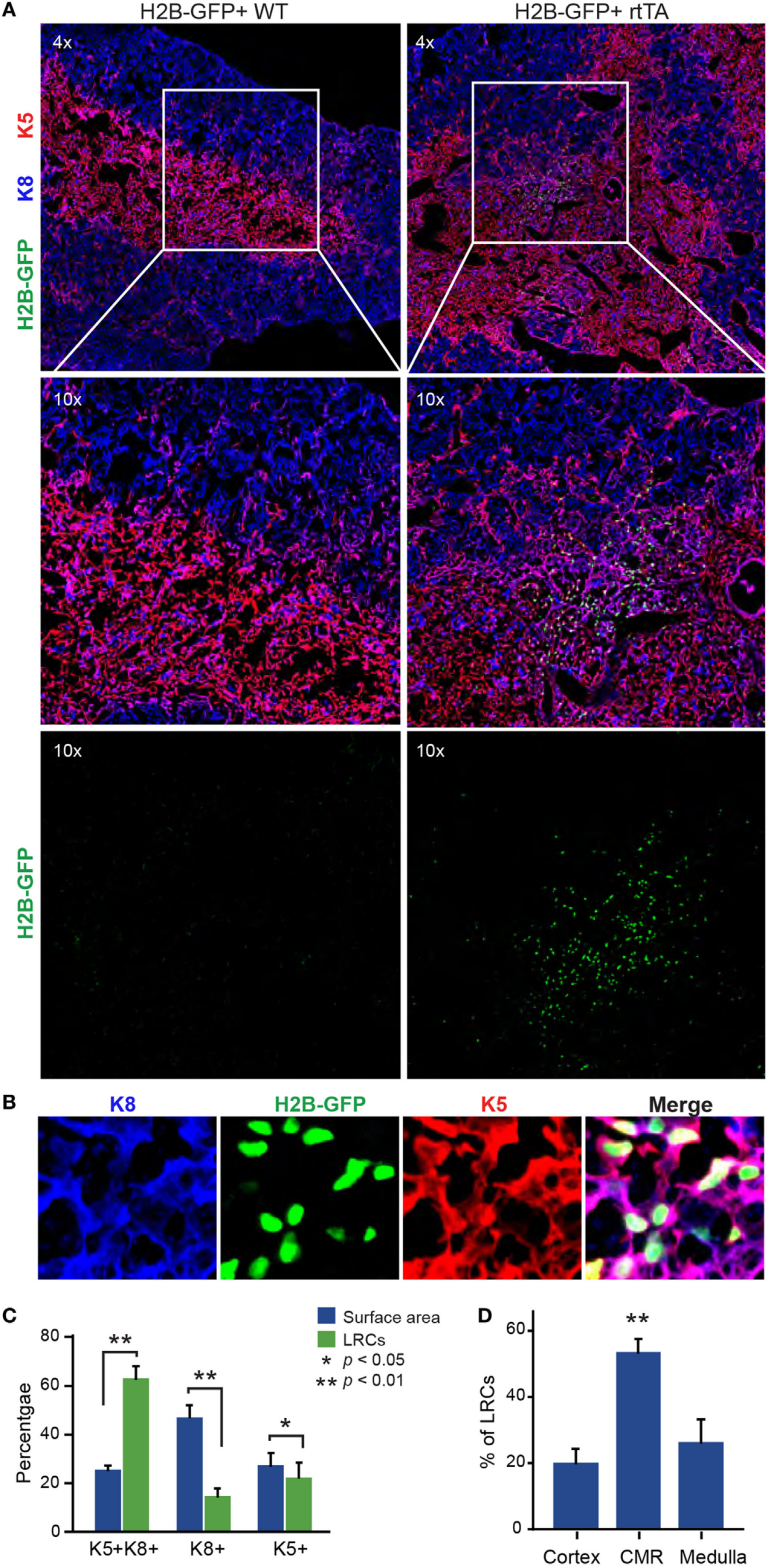
Most label-retaining cells (LRCs) are K5^+^K8^+^ and localized near the cortico-medullary junction. **(A)** Representative images of thymic slices after 16 weeks of chase in control (H2B-GFP^+^ WT, left panels) and test mice (H2B-GFP^+^ rtTA, right panels). **(B)** Representative images of K5^+^K8^+^ GFP^hi^ cells. Cytokeratin K5 is shown in red, K8 in blue, and histone 2B-GFP fusion protein (H2B-GFP) in green. **(C)** Quantification of LRCs expressing K5, K8, or both cytokeratins. The number of LRCs per thymic lobe (green) from each TEC subset is compared with the surface area (blue) covered by this subset. **(D)** Percentage of LRCs found in different regions of the thymus. The proportions of LRCs are significantly higher in the cortico-medullary region (CMR) than the cortex or the medulla (*n* = 4).

Using immunofluorescence microscopy, we first investigated the location and phenotype of LRC TECs after the 16-weeks chase period. In tissue sections, we defined TECs as cells expressing cytokeratin 8 (K8) and/or cytokeratin 5 (K5) and LRCs as cells having a fluorescence intensity four times greater than the cells with the maximal signal in the negative control (see Figure S1 in Supplementary Material). Labeling with antibodies against K8 and K5, respectively, defined cortical and medullary regions of the thymus (Figure 1A). We evaluated the number of LRCs in each TEC subset (K5^+^, K8^+^, and K5^+^K8^+^) and the relative surface area covered by individual subsets. The salient finding was that LRCs were depleted from the K8^+^ and K5^+^ population and significantly enriched in the K5^+^K8^+^ population (Figures 1B,C). Indeed, while K5^+^K8^+^ TECs only occupy 26% of the surface area, they contain 63% of the LRCs. Since expression of both K5 and K8 is typical of TEPCs at embryonic day E12.0 (33) and becomes rare in the adult thymus, we conclude that most LRCs have a phenotype typical of undifferentiated TEPCs.

**Figure 2:**
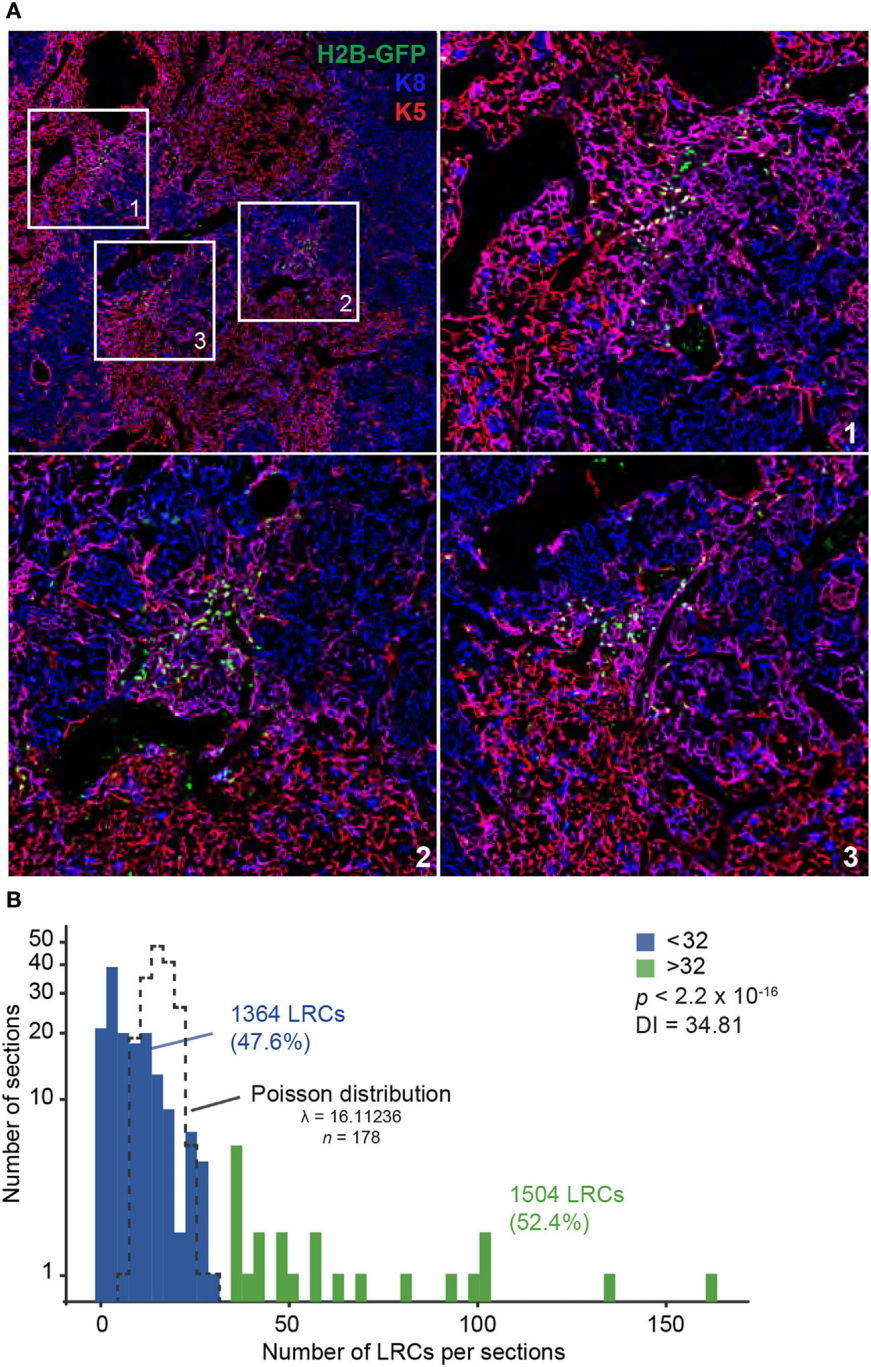
Label-retaining cells (LRCs) are found in clusters at the cortico-medullary junction (CMJ). **(A)** Representative images of LRC clusters near the CMJ. Cytokeratin K5 is shown in red, K8 in blue, and H2B-GFP in green. **(B)** LRC distribution in the thymus. Each thymic slice was divided in 40–50 sections (see Figure S3B in Supplementary Material) for LRC quantification. Histogram shows the number of sections on the *Y*-axis (projected on a log scale) and the number of LRCs per section on the *X*-axis. The TEC distribution did not fit a Poisson distribution, shown with a black dotted line (*p* < 2.2 × 10^−16^; goodness of fit, maximum likelihood).

We next counted the number of LRCs found in each region of the thymus: cortex, medulla, and cortico-medullary region (CMR). The CMR was defined as the area spanning 100 µm on either side of the CMJ delimited by K5 (Figure S3A in Supplementary Material). Notably, more than half of LRCs (53.6%) were found in the CMR (Figure 1D). We also observed that many LRCs formed clusters, while large regions of the stroma were devoid of any LRCs (Figure 2A). To quantify this phenomenon, we separated thymic slices in 40–50 non-overlapping sections of equal surface area (Figure S3B in Supplementary Material) and counted the number of LRCs expressing at least one cytokeratin in each section. While individual sections contained an average of 16 LRCs, we observed that half (52.4%) of LRCs were located within sections containing >32 LRCs, a few even containing more than 100 LRCs each (Figure 2B). However, only 12.9% of all areas contained such clusters (in green, Figure 2B). To rule out the possibility that this distribution was random, we compared it with a Poisson distribution created with the LRCs distribution’s average (λ = 16.11236) and containing the same number of values (*k* = 178) and confirmed that the distributions were significantly different (*p* < 2.2 × 10^−16^; goodness of fit). We then calculated the dispersion index associated with LRCs’ distribution. In a context where the positions of events would be completely independent of one another, the dispersion index would be equal to 1, as represented by the Poisson distribution. In a non-randomly dispersed dataset, a dispersion index > 1 means that the events tend to group together, leaving empty spaces in-between clusters of events. On the opposite, a dispersion index < 1 corresponds to a pattern of organization more regular than the randomness associated with the Poisson distribution. The dispersion index of LRCs in thymic slices was 34.81, showing that LRCs were grouped in clusters (Figure 2B). Together, these results show that most LRCs present an undifferentiated phenotype and are found in clusters at the CMJ, suggesting the existence of a specialized microenvironment (niche) for these quiescent cells.

### UEA1^–^ LRCs Increase Their Proliferation Rate following Acute Thymic Injury

**Figure 3:**
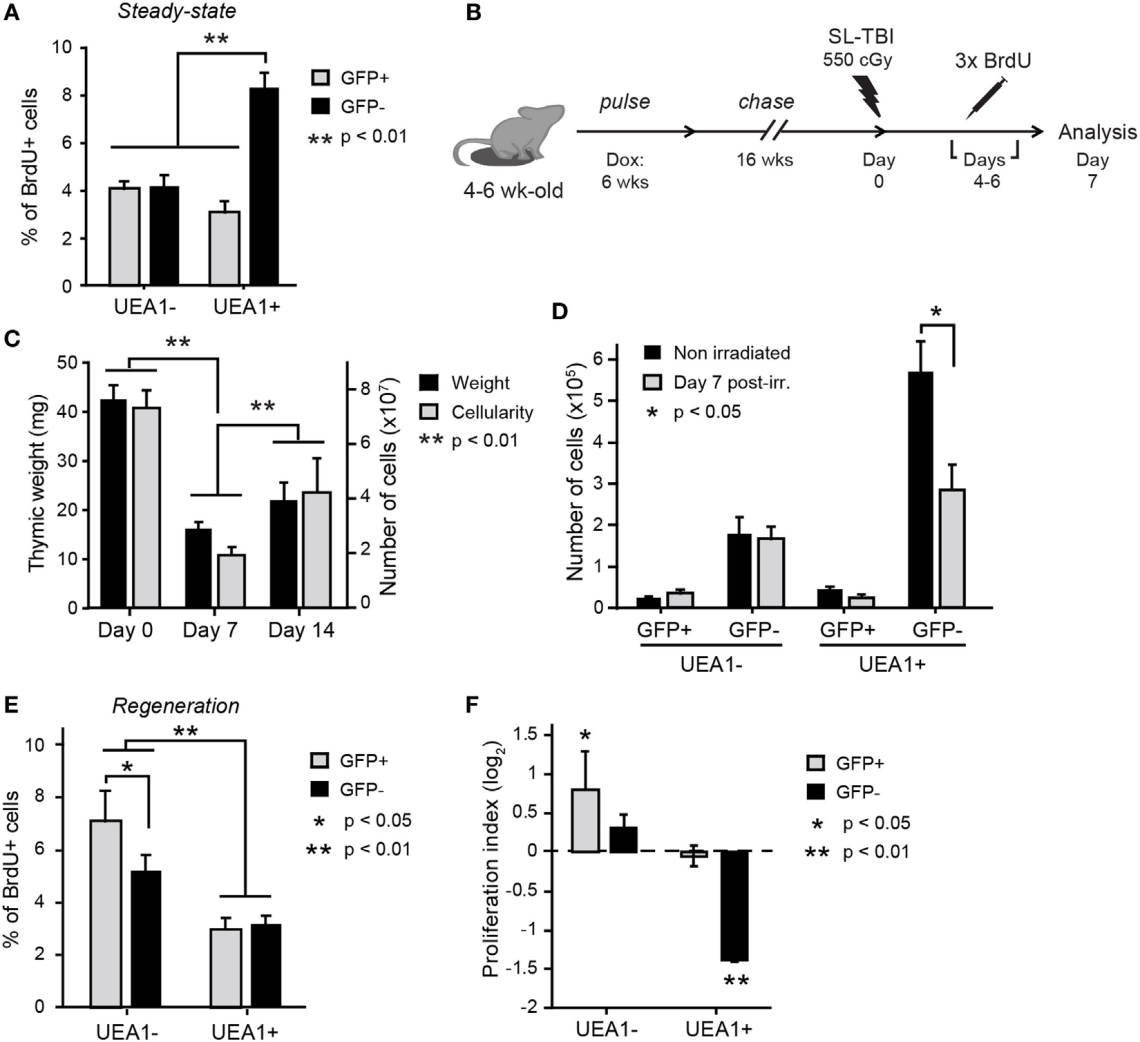
UEA1^–^ label-retaining cells (LRCs) participate to thymic regeneration following SL-TBI. **(A)** Percentage of BrdU^+^ thymic epithelial cells (TECs) under steady-state conditions. Statistical differences were calculated using paired Student’s *t*-test (*n* = 10). **(B)** Schematic representation of the thymic injury assay. **(C)** Thymic weight (black) and cellularity (gray) at different times before and after irradiation (*n* = 4–11 per group). **(D)** Number of cells in each TEC subset in non-irradiated controls (day 0, black) and during regeneration following SL-TBI (day 7, gray, *n* = 5). Percentage of BrdU^+^ TECs **(E)** and proliferation index **(F)** for each subpopulation of TECs during thymic regeneration. The proliferation index is calculated using the following equation: proportion of BrdU^+^ cells during regeneration (day 7)/proportion of BrdU^+^ cells before SL-TBI (day 0). Cells derived from LRCs (labeled GFP^+^) or NonLRCs (labeled GFP^–^) are represented in gray and black, respectively (*n* = 9–10 per group). Statistical differences for panels **(C,D,F)** were calculated by cell population, comparing day 0 non-irradiated to day 7 postirradiation or day 14 to day 7 postirradiation. Data are represented as mean + SEM.

We studied TEC proliferation using BrdU incorporation. It must be reminded that upon division, the H2B-GFP content in LRCs decreases by 50% in the daughter cells, and therefore LRCs’ progeny might become GFP^int^. Consequently, we reasoned that cells derived from LRCs which had undergone one or two cell divisions would be BrdU^+^ and either GFP^hi^ or GFP^int^ (regrouped under GFP^+^ in Figure 3). This definition may slightly underestimate the LRC progeny because after three cell divisions, the LRC progeny would become GFP^–^ in tissue sections (through dilution). To minimize this bias, we allowed BrdU incorporation during only a short pulse period of three days, starting at the end of the 16-week H2B-GFP chase period. Thymi were then extracted on the following day and analyzed for BrdU incorporation. Under steady-state conditions, most BrdU^+^ TECs derived from UEA1^+^ NonLRCs (labeled GFP^–^ in Figure 3). This is consistent with previous reports that mTECs (UEA1^+^) proliferate more extensively than other TEC subsets in adult mice (7, 25).

We next assessed TEC regeneration following acute thymic injury, using the well characterized model of sublethal-total body irradiation (SL-TBI) without hematopoietic rescue. SL-TBI induces an acute thymic involution and thymic weight reaches a nadir 3 days after irradiation (7, 34, 35). This involution phase is followed by a regenerative phase as thymic cellularity returns to normal levels within a few weeks. At the end of the H2B-GFP chase period (i.e., at 26–28 weeks of age), mice were irradiated on “day 0” with a sublethal dose of 550 cGy (Figure 3B). As expected, we observed global thymic hypocellularity 7 days after SL-TBI, followed by regeneration between day 7 and 14 (Figure 3C). Consistent with the notion that actively cycling cells are particularly sensitive to irradiation (36), the decrease in TEC numbers on day 7 post-SL-TBI was due exclusively to the loss of UEA1^+^GFP^–^ NonLRCs (Figure 3D).

To monitor TEC proliferation at the onset of thymic regeneration, mice were given daily intraperitoneal injections of BrdU from day 4 to day 6 after SL-TBI (Figure 3B), i.e., during the first days of thymic regrowth (11, 34, 35), and the percentage of BrdU^+^ cells was assessed at day 7 (Figure 3E). While BrdU incorporation can occur through both DNA repair and DNA replication, the amount of BrdU incorporated during those two events is very different, as DNA repair occurs in localized foci in the genome (37). The median fluorescence intensity (MFI) of BrdU^+^ TECs was more than 7× that of the whole cell population for both non-irradiated controls and day 7 postirradiation (Figure S4 in Supplementary Material), showing that BrdU incorporation after irradiation truly results from DNA replication. Also, BrdU MFI higher than that of BrdU^+^ TECs in the non-irradiated control (Figure S4 in Supplementary Material), suggesting that cells proliferating after irradiation went through more cycles of proliferation than those proliferating in steady-state. Interestingly, when compared with steady-state conditions (Figure 3A), the proportion of BrdU^+^ cells during post-SL-TBI regeneration showed conspicuous changes in two TEC subsets. First, the frequency of BrdU^+^ cells among UEA1^+^ NonLRCs (GFP^–^) was decreased by more than twofold (Figures 3E,F). Second, the salient finding was a conspicuous increase in the proportion of BrdU^+^UEA1^–^ LRCs which surpassed the proportion of BrdU^+^ elements in all other TEC subsets (Figures 3E,F). Overall, these results show that (i) UEA1^+^ NonLRCs are greatly affected by irradiation, resulting in cell death and decreased proliferation and (ii) that UEA1^–^ LRCs enrich for radioresistant TEPCs that actively proliferate during tissue regrowth.

### Cell Surface Phenotype of UEA1^–^ LRCs

**Figure 4:**
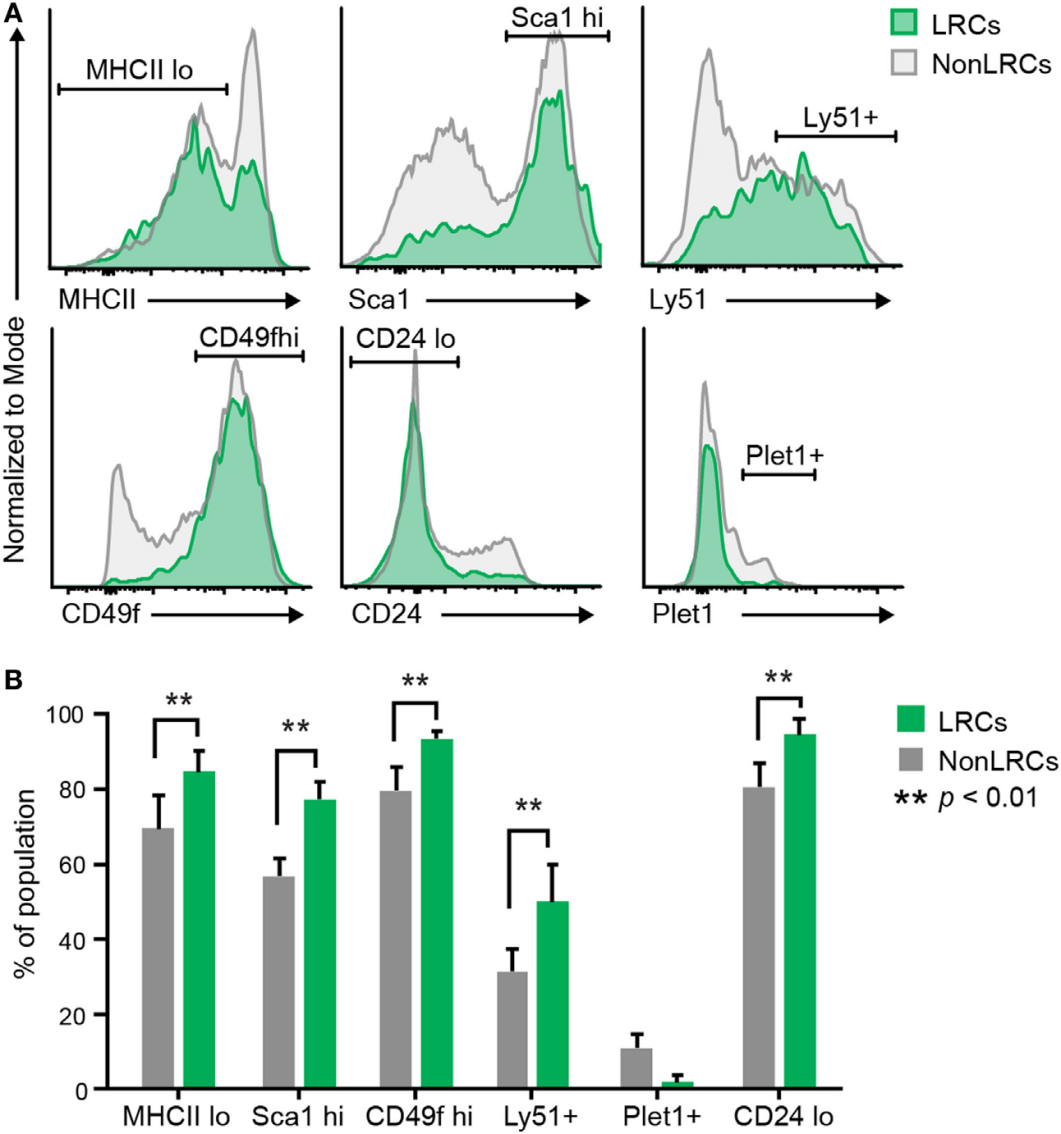
Cell surface markers of UEA1^–^ label-retaining cells (LRCs). **(A)** Representative expression of cell surface markers on UEA1^–^ thymic epithelial cells. LRCs are represented in green and NonLRCs in gray. **(B)** Proportion of UEA1^–^ LRCs and NonLRCs displaying specified phenotypes (*n* = 2–9 per marker).

Recent studies have reported the presence of bipotent TEPCs in the adult thymus. However, the cell surface phenotype of bipotent TEPCs in these three reports showed significant discrepancies (38–40). We therefore assessed whether UEA1^–^ LRCs expressed cell surface markers previously reported in bipotent TEPCs (Table S2 in Supplementary Material). When compared with UEA1^–^ NonLRCs, UEA1^–^ LRCs showed increased proportion of MHCII^lo^, Sca1^hi^, Cd49f^hi^, and Ly51^+^ elements (Figure 4). Furthermore, UEA1^–^ LRCs were EpCAM^+^ (Figure S2 in Supplementary Material) and Plet1^–^ (Figure 4B). Therefore, the phenotype of UEA1^–^ LRCs is remarkably similar to that of TEPCs reported by Wong et al. which were EpCAM^+^UEA1^–^MHCII^lo^Sca1^hi^ and CD49f^hi^ Plet1^–^ (38).

### Transcriptomic Analysis of UEA1^–^ LRCs and NonLRCs

To gain insights into the molecular and functional attributes of UEA1^–^ LRCs (enriched in radioresistant TEPCs), we compared their transcriptome to that of UEA1^–^ NonLRCs. To this end, we extracted and sequenced poly-A enriched mRNAs from sorted UEA1^–^ LRCs and NonLRCs using the Illumina HiSeq2000 platform. A total of 2,078 genes showed differential expression (fold change >2) between UEA1^–^ LRCs and NonLRCs, of which 1,450 (69.8%) were downregulated in UEA1^–^ LRCs (Table S3 in Supplementary Material). We then analyzed GO-term enrichment for each of the top 500 most differentially expressed genes. Using the gene-annotation enrichment tool DAVID (21, 22), we extracted GO-terms for each cell population and used the REViGO software to reduce GO-terms redundancy.

**Figure 5:**
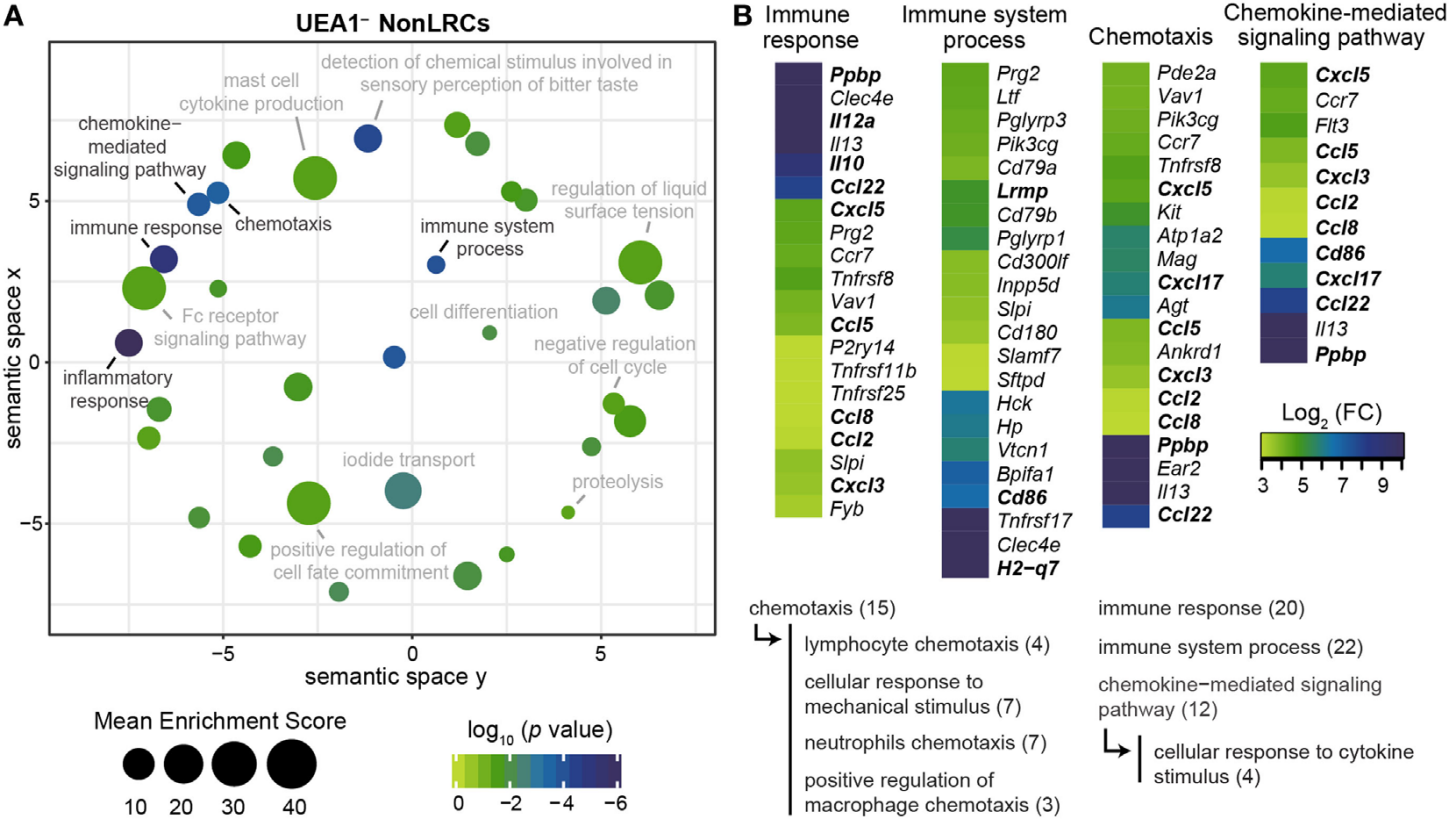
UEA1^–^ NonLRCs are well adapted to communicate with hematopoietic cells. **(A)** REViGO graphical representation of significant gene ontology (GO)-term enrichment for genes expressed at higher level in UEA1^–^ NonLRCs than UEA1^–^ label-retaining cells (LRCs). The position of GO-terms represents their semantic similarities calculated by REViGO, the size represents the mean enrichment score of the GO-terms contained in each point, and the color indicates the *p* value for that particular GO-term. **(B)** Heatmap showing the differential expression of genes involved in GO-terms related to interactions with hematopoietic cells. All GO-terms are determined using DAVID online bioinformatic gene enrichment tool from the top 500 genes expressed at higher level in UEA1^–^ NonLRCs than UEA1^–^ LRCs. Semantic relations between GO-terms of interest are shown below, and the number of genes per GO-term is shown in parentheses.

Four GO-terms associated with the regulation of thymocytes maturation by TECs were significantly enriched in the gene set upregulated in UEA1^–^ NonLRCs (Figure 5). Notably, genes involved in antigen presentation (*Lrmp* and *H2-q7*) and thymocyte stimulation (*Cd86, Il10*, and *Il12a*) and chemotaxis (*Ccl22* and *Ppbp*) were upregulated in NonLRCs (Figure 5B) (41–43). Moreover, many chemokines involved in the chemotaxis of dendritic cells and macrophages (*Ccl2, Ccl5, Ccl8, Cxcl3, Cxcl5*, and *Cxcl17*) were also upregulated in UEA1^–^ NonLRCs (44, 45). These results suggest that UEA1^–^ NonLRCs are better equipped than UEA1^–^ LRCs to interact with hematopoietic cells.

**Figure 6:**
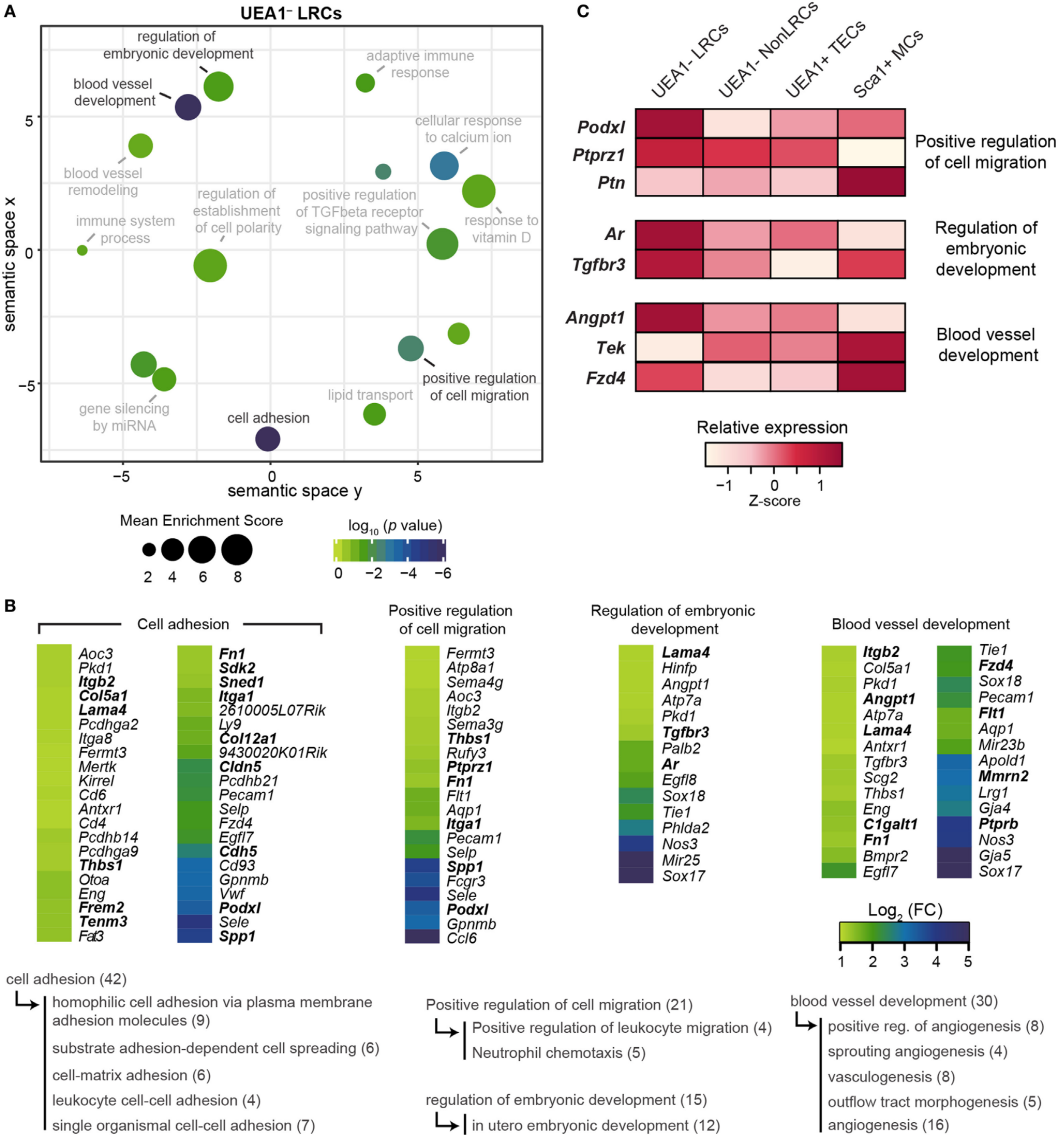
Transcriptomic analysis of UEA1^–^ label-retaining cells (LRCs) reveals potential regulators of thymic epithelial progenitor cells (TEPCs). **(A)** REViGO graphical representation of semantic relations between gene ontology (GO)-terms related to the top 500 genes expressed at higher level in UEA1^–^ LRCs than UEA1^–^ NonLRCs. The position of GO-terms represents their semantic similarities calculated by REViGO, the size represents the mean enrichment score of the GO-terms contained in each point, and the color indicates the *p* value. **(B)** Heatmap showing the differential expression of genes responsible for GO-term enrichment in panel **(A)**. Semantic relations between GO-terms are shown below, with the number of genes shown in parenthesis. **(C)** Relative expression (*Z*-score) of potential regulators of TEPCs in thymic stromal cell subsets.

On the other hand, several genes upregulated in UEA1^–^ LRCs were associated with cell adhesion and cell migration (Figures 6A,B), including many secreted ECM proteins (*Col12a1, Col5a1, Fn1, Frem2, Lama4*, and *Sned1*) and molecules involved in cell–cell or cell–matrix adhesion (*Cldn5, Cdh5, Itga1, Itgb2, Sdk2, Tenm3*, and *Thbs1*). Contrary to UEA1^–^ NonLRCs, UEA1^–^ LRCs therefore seemed more adapted to interact with the surrounding stromal cells and ECM. Two genes specifically upregulated in UEA1^–^ LRCs are involved in the regulation of stem cell activity in several tissues: *Podxl* and *Ptprz1* (Figure 6C). *Podxl*, a marker of cardiac, hematopoietic and mesenchymal stem cells, is involved in the maintenance of the immature state in cardiac stem cells and its downregulation facilitates their differentiation (46–48). *Ptprz1* is expressed in human embryonic stem cells and is downregulated during differentiation. While PTPRZ1 depletion is associated with a decrease in colony-formation potential of embryonic stem cells, its activation enhances the proliferation of embryonic stem cells (49). PTPRZ1 also negatively regulates oligodendrocyte precursor proliferation (50). Interestingly, the *Ptprz1* ligand *Ptn*, expressed by fibroblasts in the embryo, is also highly expressed by Sca1^+^ thymic mesenchymal cells (Figure 6C) but absent in TECs. The expression profile of *Ptprz1* (by UEA1^–^ LRCs) and *Ptn* (by Sca1^+^ mesenchymal cells) suggests that Sca1^+^ mesenchymal cells may be key components of the TEPC niche, as observed in other tissues (24). From these results, we conclude that *Podxl* and *Ptprz1* represent potential regulators of TEPC maintenance *via* interactions with stromal niche components.

Another GO-term significantly enriched in UEA1^–^ LRCs is directly related to stem cell regulation: *regulation of embryonic development* (Figures 6A,B). Two pathways associated with this GO-term are also known to regulate thymopoiesis: androgen receptor and TGF-beta. Androgen receptor is expressed by TECs and regulates their proliferation. Indeed, administration and ablation of androgen, respectively, lead to acute thymic involution and hypertrophy (6, 12, 13, 51). Of note, expression of androgen receptors by stromal cells is necessary for androgens to affect thymic cellularity (13, 51, 52). The fact that UEA1^–^ LRCs express higher levels of *Ar* transcripts than other types of thymic stromal cells (Figure 6C) suggests that the impact of androgens on thymopoiesis could be mediated primarily *via* regulation of TEPCs. In addition, TGF-beta signaling in TECs was previously shown to decrease mTEC differentiation and maturation (53). Hence, though the precise role of TGFBR3 in TECs has not been elucidated, its higher expression in UEA1^–^ LRCs suggests that it might regulate their activity or differentiation (Figure 6C).

Typically, stem cells preferentially reside close to blood vessels in many tissues, including the bone marrow, hair follicle bulge and testes (54–56). Interestingly, the GO-term blood vessel development showed a very significant enrichment in UEA1^–^ LRCs (*p* < 10^−7^, Figures 6A,B) and several genes overexpressed in UEA1^–^ LRCs regulate blood vessel stability (*Angpt1, Ptprb, C1galt1, Lama4, Mmrn2*, and *Flt1*). Interestingly, in addition to its implication in blood vessel development *Angpt1* is also involved in hematopoietic stem cell maintenance. Hematopoietic stem cells express high levels of ANGPT1, and TEK-ANGPT1 signaling facilitates adhesion of hematopoietic stem cells to their niche, increasing their stem cell activity (57). Of note, thymic Sca1^+^ mesenchymal cells also express high levels of *Tek*, the ligand for *Angpt1* (Figure 6C), suggesting that TEK-ANGPT1 interaction might also be involved in the maintenance of TEPCs. Finally, the WNT4 receptor *Fzd4* was expressed at higher levels in UEA1^–^ LRCs and Sca1^+^ mesenchymal cells compared with other stromal populations (Figure 6C). As WNT4 signaling enhances thymic cellularity through the expansion of TECs (58) and decreased WNT4 production contributes to thymic involution (59), expression of *Fzd4* by the TEPC-enriched population of UEA1^–^ LRCs and by Sca1^+^ mesenchymal cells suggests that WNT4 may regulate TEC maintenance primarily by regulating these two cell populations. Overall, our transcriptomic analyses suggest that while UEA1^–^ NonLRCs are specialized to communicate with hematolymphoid cells, UEA1^–^ LRCs interact mostly with the surrounding stromal cells and ECM. Furthermore, we have identified through transcriptomic sequencing six potential regulators of TEPCs (*Podxl, Ptprz1, Ar, Tgfbr3, Angpt1*, and *Fzd4*) that are upregulated in UEA1^–^ LRCs.

## Discussion

In a previous study, we have demonstrated the presence of non-senescent LRCs in UEA1^–^ TECs (7). We now report that, together with their quiescent state, their resistance to radiations and their undifferentiated phenotype (K5^+^K8^+^) the ability of UEA1^–^ LRCs to participate to TEC regeneration after acute injury qualifies them as TEPCs. This conclusion is strengthened by two features of our experimental design. First, because of the constraints of the label-retention assay, our analyses on UEA1^–^ LRCs were performed in mice that were quite old, at 26–28 weeks of age. At this age, one would expect the global TEC population to have a limited proliferation potential. Indeed, previous studies of TEPCs were performed either in newborns or in 6–8 weeks old mice (38–40). Second, we analyzed the proliferation of UEA1^–^ LRCs *in situ*, without transplantation nor any *in vitro* purification step or culture. It has been shown that cells which are extracted from their normal environment and transplanted can acquire stem cell properties that they do not display under steady-state conditions (19, 60). We therefore conclude that even in relatively old mice, UEA1^–^ LRCs have genuine TEPC activity after acute involution. During the regenerative phase following acute thymic injury induced by SL-TBI, UEA1^–^ LRCs were the most actively proliferating TEC subset. Interestingly, while the UEA1^–^ LRCs did not proliferate much under steady-state conditions, they increased their proliferation in the context of tissue repair. On the other hand, the TECs which proliferate most under steady-state conditions, i.e., UEA1^+^ NonLRCs, are the most affected by irradiation and barely proliferate during thymic regeneration. Of note, in addition to cell-intrinsic features (i.e., high percentage of cycling elements), the exquisite sensitivity of thymic UEA1^+^ NonLRCs to acute injury may be in part cell-extrinsic. Indeed, thymocyte-TEC cross talk is involved in thymic regeneration following total body irradiation (61). As irradiation leads to the depletion of thymocytes, it might disrupt interactions that allowed sustained proliferation and survival in mTECs. As UEA1^–^ LRCs express lower levels of genes involved in TEC-thymocyte interactions (Figure 6), they might be less dependent on cross talk with thymocytes. Our observations on TEC turnover are reminiscent of the intestinal epithelium, which possesses two types of stem cells: (i) rapidly cycling LGR5^+^ stem cell, responsible for steady-state maintenance of the intestinal epithelium and (ii) a pool of reserve quiescent stem cells that can compensate when the rapidly cycling LGR5^+^ stem cells are damaged (62). Our data suggest that UEA1^–^ LRCs are similar to the reserve quiescent stem cells in the intestinal epithelium. Likewise, other tissues including the cornea and the bone marrow contain quiescent stem cells that can be called upon in a context of injury (60, 62–64).

UEA1^–^ LRCs shared two features with the bipotent progenitors identified by Wong et al.: they display similar cell surface phenotypes and are both LRCs (38). This suggests that UEA1^–^ LRCs might contribute to the regeneration of both cTECs and mTECs following irradiation-induced injury. However, Ohigashi et al. reported that in adults, cTECs and mTECs were maintained by distinct progenitors, distinguished by their expression of the β5T immunoproteasome subunit (65). Therefore, it is also possible that UEA1^–^ LRCs contribute only to the regeneration of cTECs. Our model unfortunately has limitations, inherent to the nature of the label-retention assay, which prevents us to fully explore these hypotheses. First, UEA1^–^ LRCs were enriched in TEPCs but they did not represent a pure TEPC population. Second, label-retention assays do not allow to perform *in situ* lineage tracing of LRCs to precisely characterize their differentiation potential. Pure quiescent TEPC populations and lineage tracing assays will be necessary to properly address these issues, which will require discovery and validation of new quiescent TEPC-specific markers.

Nevertheless, in addition to the identification of TEPCs in UEA1^–^ LRCs, our work (i) shows that most UEA1^–^ LRCs are found in clusters in the vicinity of the CMJ and (ii) presents a systems-level transcriptomic analysis of this TEPC-enriched cell subset. Moreover, RNA-Seq analyses revealed several interesting features of UEA1^–^ LRCs. Based on their transcriptome, UEA1^–^ NonLRCs would appear more qualified to interact with thymocytes whereas UEA1^–^ LRCs seem more adapted to interactions with stromal cells and the ECM. These findings, together with evidence that UEA1^–^ LRCs are preferentially located in clusters in the CMR, suggest the existence of a specialized niche for TEPCs. More specifically, we identified three genes that may play a role in the regulation of TEPCs through interactions with niche cells: *Ptprz1, Podxl*, and *Angpt1*. In particular, the expression of the *Ptprz1* ligand *Ptn* by Sca1^+^ mesenchymal cells suggest that these cells are important components of the TEPC niche, as they are for the hematopoietic stem cell niche (24). Furthermore, many genes involved in blood vessel development were upregulated in UEA1^–^ LRCs compared with NonLRCs. This observation is coherent with the fact that blood vessels are crucial components of many types of stem/progenitor cell niches (66). Finally, among genes expressed at higher levels in UEA1^–^ LRCs than in UEA1^–^ NonLRCs, we identified three receptors whose ligands regulate TEC homeostasis: *Ar, Fzd4*, and *Tgfrb3*. Identification of these potential TEPC regulators warrants further investigation to better understand the mechanisms regulating the function of TEPCs and their interactions with niche cells. Investigations along these lines could provide evidence based strategies for enhancing TEC regeneration.

## Ethics Statement

All procedures were in accordance with the Canadian Council on Animal Care guidelines and approved by the Comité de Déontologie et Expérimentation Animale de l’Université de Montréal.

## Author Contributions

MD-L: conception and design, collection, assembly, analysis, and interpretation of data, and manuscript writing. HG: conception and design, collection and assembly of data, and final revisions of the manuscript. TD, J-PL, SB, and SL: analysis and interpretation of data and final revisions of the manuscript. EG: conception and design and manuscript writing. CP: conception and design, provision of study material, financial support, and manuscript writing.

## Conflict of Interest Statement

The authors declare that the research was conducted in the absence of any commercial or financial relationships that could be construed as a potential conflict of interest.

## Footnotes

^1^https://www.ncbi.nlm.nih.gov/geo/query/acc.cgi?acc=GSE94642.

^2^http://www.r-project.org/.

^3^https://david.ncifcrf.gov/.

## Supplementary Material

Table S1 Antibodies used for immunofluorescence microscopy (IFM) and flow cytometry (FC) analyses.

Table S2 Comparison of the cell surface phenotype of UEA1–LRCs with that of TEPCs identified in other reports. Features shared with UEA1– LRCs are indicated in bold green.

Table S3 Gene expression in UEA1–LRCs and Non-LRCs assessed by RNA-sequencing.
