# Supplementary material for: Detection of Quiescent Radioresistant Epithelial Progenitors in the Adult Thymus
Source: Front Immunol. 2017 Dec 5;8:1717. doi: 10.3389/fimmu.2017.01717 (PMC5723310; doi:10.3389/fimmu.2017.01717)
Supplement: Table S1 — Antibodies used for immunofluorescence microscopy (IFM) and flow cytometry (FC) analyses. [file Table_1.docx]

**Supplementary Table S1 -** Antibodies used for immunofluorescence microscopy (IFM) and flow cytometry (FC) analyses.

| **Antigen** | **Clone** | **Supplier** | **Staining** |
| --- | --- | --- | --- |
| EpCAM - APC-Cy7 | G8.8 | BioLegend | FC |
| CD45 - PE-Cy5 | 30-F11 | BD Biosciences | FC |
| CD45 - Alexa Fluor 700 | 30-F11 | BD Biosciences | FC |
| UEA1 - biotinylated | Purified lectin | Vector Laboratories | FC |
| I-A/I-E - Alexa Fluor 700 | M5/114 15.2 | BioLegend | FC |
| I-A/I-E - APC | M5/114 15.2 | BioLegend | FC |
| Ly-6A/E (Sca1) - PerCP-Cy5.5 | D7 | eBioscience | FC |
| CD49f (α6-integrin) - APC | GoH3 | eBioscience | FC |
| CD49f (α6-integrin) - PE-Cy7 | GoH3 | eBioscience | FC |
| CD24 - PE | M1/69 | BD Biosciences | FC |
| CD24 - PE-Cy7 | M1/69 | BD Biosciences | FC |
| CD24 - Alexa Fluor 700 | M1/69 | BD Biosciences | FC |
| BrdU flow kit - APC | Kit | BD Biosciences | FC |
| Cytokeratin 8 - purified | TROMA-I | DSHB Iowa | IFM |
| Keratin 5 polyclonal- purified | Poly19055 | BioLegend | IFM |
| Donkey anti-rabbit IgG – DyLight 405 | Poly4054 | BioLegend | IFM |
| Goat anti-rat IgG – DyLight 594 | Poly4064 | BioLegend | IFM |
| Streptavidin – PE-Cy7 | - | BD Biosciences | FC |
| Streptavidin – PE-Texas Red | - | BD Biosciences | FC |
| Plet1 – Purified | ID4 | EMD Millipore | FC |
| Ly51 – Alexa Fluor 647 | 6C3 | Biolegend | FC |
