# Supplementary material for: Detection of Quiescent Radioresistant Epithelial Progenitors in the Adult Thymus
Source: Front Immunol. 2017 Dec 5;8:1717. doi: 10.3389/fimmu.2017.01717 (PMC5723310; doi:10.3389/fimmu.2017.01717)
Supplement: Table S2 — Comparison of the cell surface phenotype of UEA1–LRCs with that of TEPCs identified in other reports. Features shared with UEA1– LRCs are indicated in bold green. [file Table_2.docx]

**Supplementary Table S2:** Comparison of the cell surface phenotype of UEA1^–^ LRCs with that of TEPCs identified in other reports. Features shared with UEA1^–^ LRCs are indicated in bold green.

**
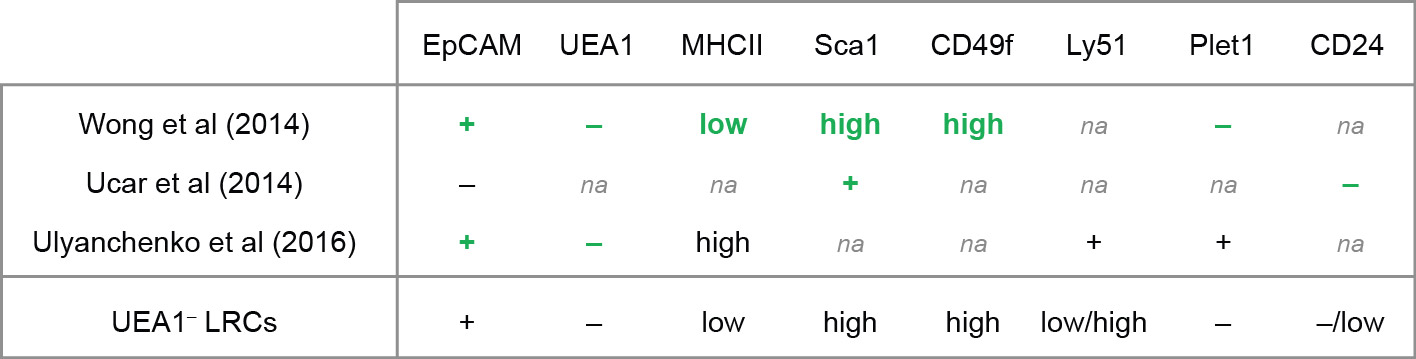
**
