# Supplementary material for: Detection of Quiescent Radioresistant Epithelial Progenitors in the Adult Thymus
Source: Front Immunol. 2017 Dec 5;8:1717. doi: 10.3389/fimmu.2017.01717 (PMC5723310; doi:10.3389/fimmu.2017.01717)
Supplement: Supplementary file 4 [file Image_1.PDF]

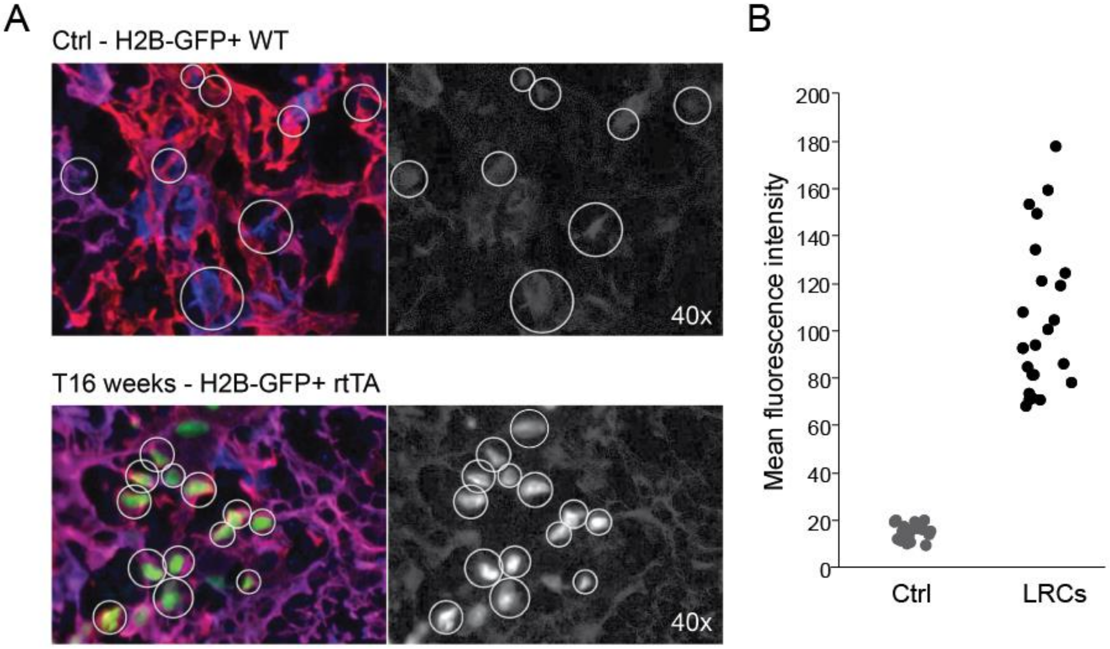

Supplementary Figure 1. Identification of LRCs on thymic slices. **a,** Representative images of thymic slices for negative control (upper panels) and T16 weeks thymic slices (lower panels). Staining for K5 (red), K8 (blue) and H2B-GFP (green) are shown on left panels and H2B-GFP fluorescence only is shown in the right panels. Circles indicate cells for which mean fluorescence intensity was measured in the negative control (i.e. cells with the highest fluorescence intensity) and examples of LRCs in the T16 weeks samples. LRCs were identified using a threshold of fluorescence four times greater than the average fluorescence intensity of the negative control. **b,** Mean fluorescence intensity in negative control and for LRCs in T16 weeks.
