# Supplementary material for: Detection of Quiescent Radioresistant Epithelial Progenitors in the Adult Thymus
Source: Front Immunol. 2017 Dec 5;8:1717. doi: 10.3389/fimmu.2017.01717 (PMC5723310; doi:10.3389/fimmu.2017.01717)
Supplement: Supplementary file 5 [file Image_2.PDF]

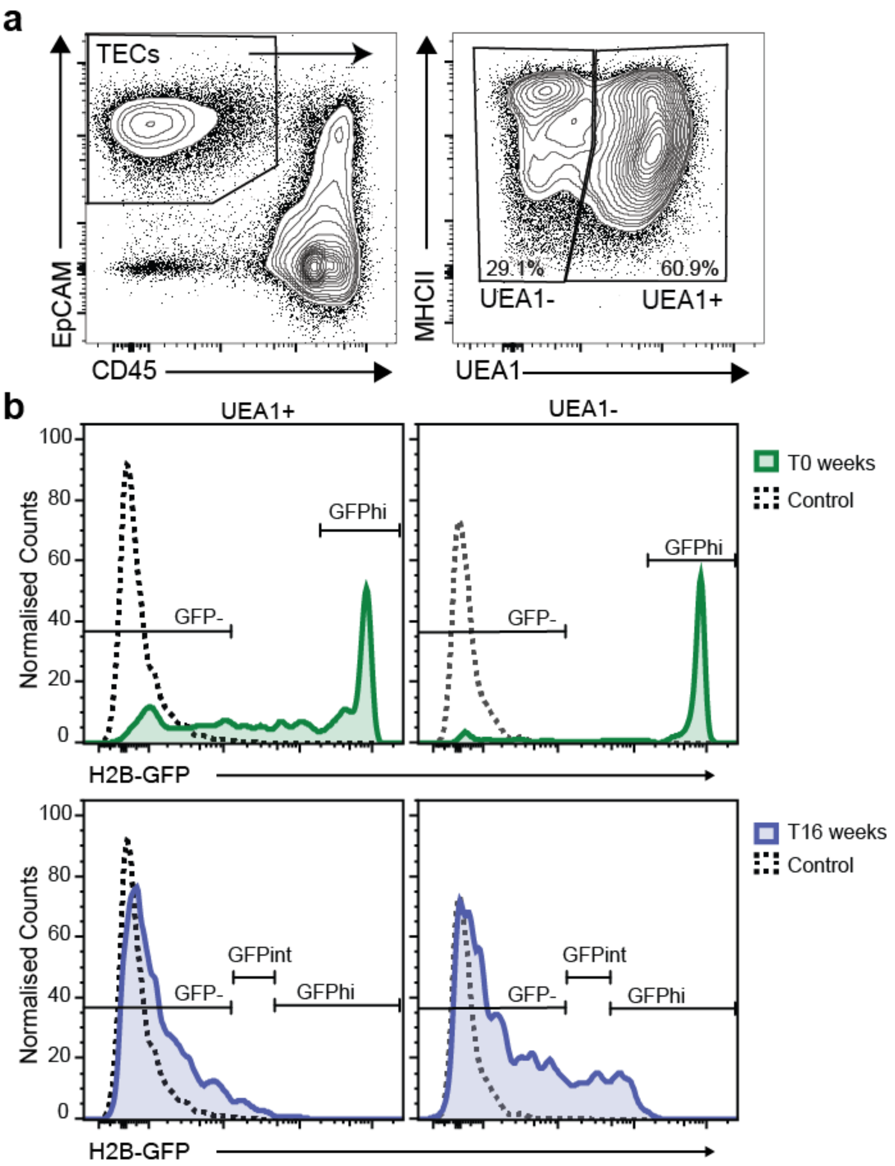

c

	T0 weeks		T16 weeks	
	GFP^{-}	GFP^{hi}	GFP^{-}	GFP^{hi}
UEA1^{-}	9.4 ± 5.1	87.8 ± 5.9	88.2 ± 4.3	5.5 ± 2.3
UEA1^{+}	19.5 ± 11.9	71.7 ± 13.5	95.3 ± 1.8	1.8 ± 1.0

Supplementary Figure 2. Gating strategy for GFP^{-}, GFP^{int} and GFP^{hi} TECs. **a**, TECs (EpCAM^{+} CD45^{-}) were separated into UEA1^{+} and UEA1^{-} subsets. **b**, The H2B-GFP fluorescence reached a maximum at the end of the pulse period (upper panels, shown in green, T0 weeks) and decreased progressively during the chase period (lower panels, shown in blue, T16 weeks). The negative control (dotted line, H2B-GFP^{+} WT) was used to define the limit between GFP^{-} and GFP^{int}. The GFP^{hi} threshold was 4x above that for GFP^{int}. **c**, Percentages of GFP^{-} and GFP^{hi} TECs in the UEA1^{+} and UEA1^{-} subsets at T0 and T16 weeks.
