# Supplementary material for: Detection of Quiescent Radioresistant Epithelial Progenitors in the Adult Thymus
Source: Front Immunol. 2017 Dec 5;8:1717. doi: 10.3389/fimmu.2017.01717 (PMC5723310; doi:10.3389/fimmu.2017.01717)
Supplement: Supplementary file 6 [file Image_3.PDF]

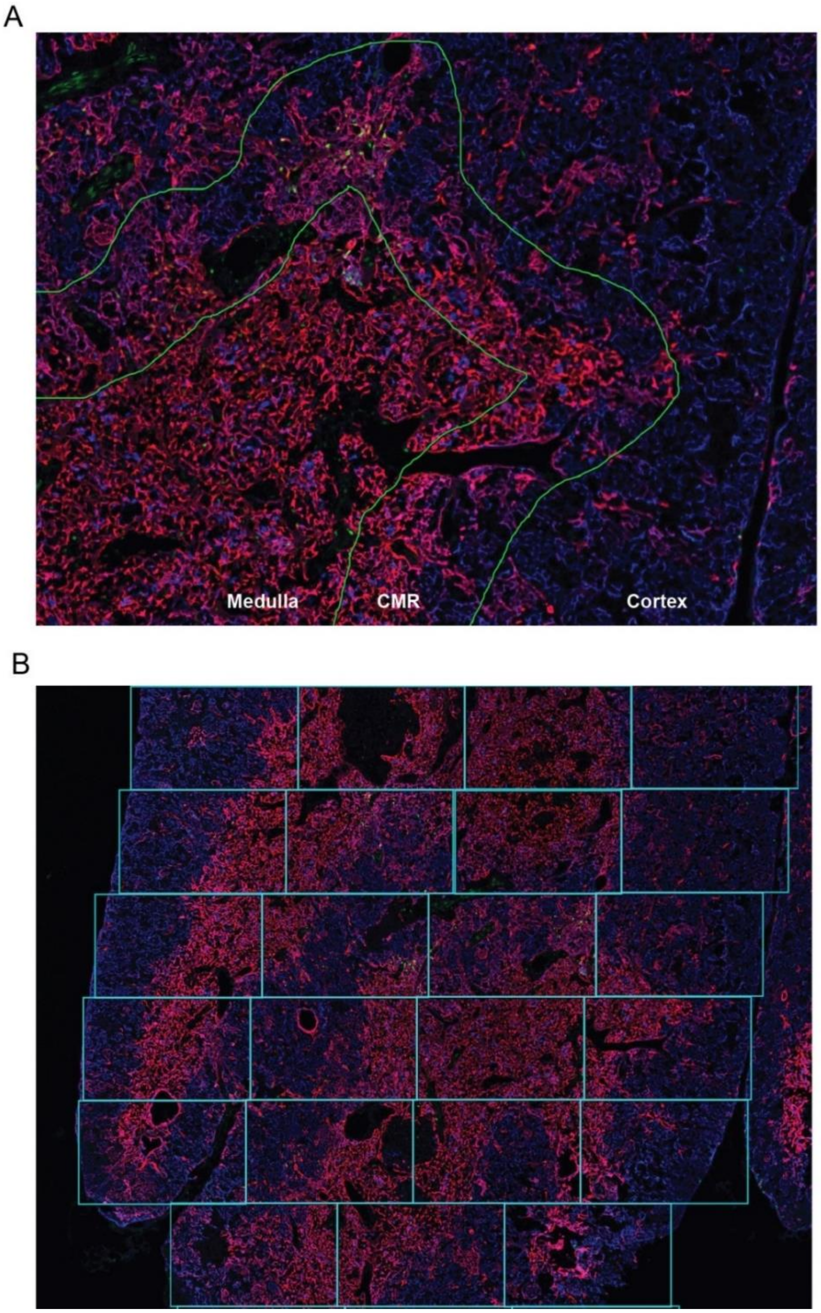

Supplementary Figure 3: Histological analysis of GFP^{+} TECs. **a,** Representative image of the cortico-medullary region, delimited in green (related to Figure 1). **b,** Representative image of a thymic slice separated into sections. Each thymic slice was separated in 40 to 50 non-overlapping sections and a total of 4 slices from 2 mice were analyzed (related to Figure 2).
