# Supplementary material for: Detection of Quiescent Radioresistant Epithelial Progenitors in the Adult Thymus
Source: Front Immunol. 2017 Dec 5;8:1717. doi: 10.3389/fimmu.2017.01717 (PMC5723310; doi:10.3389/fimmu.2017.01717)
Supplement: Supplementary file 7 [file Image_4.PDF]

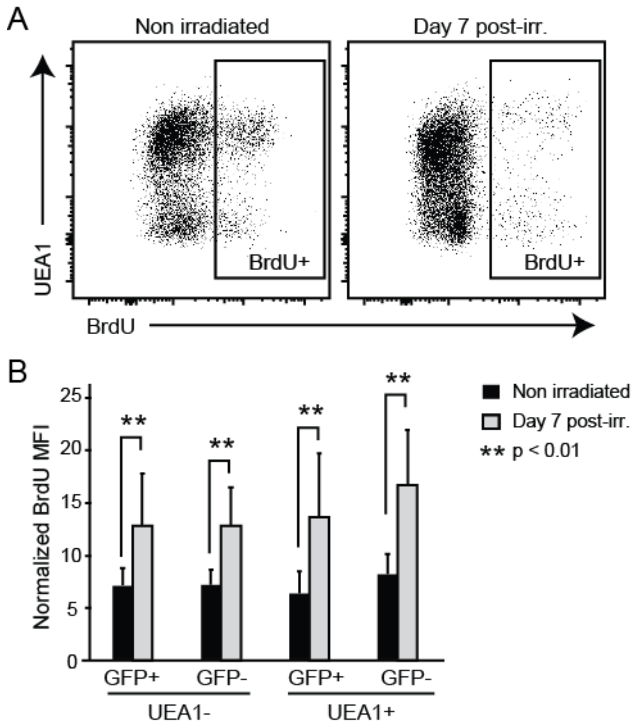

Supplementary Figure 4: BrdU incorporation in proliferating TECs is higher on day 7 post-irradiation than in non-irradiated controls. **a,** Representative staining for UEA1 and BrdU in TECs from non-irradiated mice and at day 7 post-irradiation. **b,** MFI of $BrdU^+$ TECs normalized on the average of $UEA1^-$ or $UEA1^+$ population (n = 10 to 11, representative of 3 experiments).
